# Distinct biological effects of low-dose radiation on normal and cancerous human lung cells are mediated by ATM signaling

**DOI:** 10.18632/oncotarget.12379

**Published:** 2016-09-30

**Authors:** Guozi Yang, Dehai Yu, Wei Li, Yuguang Zhao, Xue Wen, Xinyue Liang, Xiaoying Zhang, Lei Zhou, Jifan Hu, Chao Niu, Huimin Tian, Fujun Han, Xiao Chen, Lihua Dong, Lu Cai, Jiuwei Cui

**Affiliations:** ^1^ Cancer Center, The First Hospital of Jilin University, Changchun 130021, China; ^2^ Department of Radiation-Oncology, The First Hospital of Jilin University, Changchun 130021, China; ^3^ Kosair Children's Hospital Research Institute, Departments of Pediatrics, Radiation Oncology, Pharmacology and Toxicology, University of Louisville, Louisville, KY 40292, USA

**Keywords:** low-dose radiation, normal lung cells, lung cancer cells, biological effects, ATM

## Abstract

Low-dose radiation (LDR) induces hormesis and adaptive response in normal cells but not in cancer cells, suggesting its potential protection of normal tissue against damage induced by conventional radiotherapy. However, the underlying mechanisms are not well established. We addressed this in the present study by examining the role of the ataxia telangiectasia mutated (ATM) signaling pathway in response to LDR using A549 human lung adenocarcinoma cells and HBE135-E6E7 (HBE) normal lung epithelial cells. We found that LDR-activated ATM was the initiating event in hormesis and adaptive response to LDR in HBE cells. ATM activation increased the expression of CDK4/CDK6/cyclin D1 by activating the AKT/glycogen synthase kinase (GSK)-3β signaling pathway, which stimulated HBE cell proliferation. Activation of ATM/AKT/GSK-3β signaling also increased nuclear accumulation of nuclear factor erythroid 2-related factor 2, leading to increased expression of antioxidants, which mitigated cellular damage from excessive reactive oxygen species production induced by high-dose radiation. However, these effects were not observed in A549 cells. Thus, the failure to activate these pathways in A549 cells likely explains the difference between normal and cancer cells in terms of hormesis and adaptive response to LDR.

## INTRODUCTION

Conventional radiation therapy with daily doses of 1–2 Gy is a well-established and effective form of cancer treatment. Radiation is intended to deliver a sufficiently lethal dose to the target volume to achieve local tumor control while minimizing harmful effects to normal tissues so as to minimize treatment-related acute side effects and morbidity [1]. However, the success of radiation therapy is limited by systemic and normal tissue toxicity despite significant advances in medical physics. Therefore, new therapeutic strategies that not only protect normal tissue against the damage of radiation therapy but also increase the radiation sensitivity of cancer cells are urgently needed.

Accumulating evidence suggests that low-dose radiation (LDR) usually defined as ≤ 0.2 Gy at low linear energy transfer (LET) or ≤ 0.05 Gy at high LET can induce hormesis [2], which is linked to cell growth, increased longevity and embryo production, and enhanced immune response to disease [3, 4]. LDR also induces adaptive response, which protects cell and tissue against injury caused by a subsequent high-dose radiation (HDR) [5]. As a special expression of hormesis, adaptive responses are closely linked to hormetic phenomena. In other words, some of the stimulatory effects may result in adaptive response to subsequent HDR, while hormesis is the first response after exposure to adaptive response doses of radiation.

While this phenomenon has not been universally observed in all cell lines, it has been demonstrated to occur in sufficient frequency in cultured normal cells, not in many types of cancer cells. We previously showed that LDR stimulates proliferation in rat mesenchymal stem cells and mouse bone marrow hematopoietic progenitor cells, as well as in several normal human cell lines (e.g., MRC-5, HL-7702, 293T, and 6550HLEPic) [6, 7], but not in cancer cell lines (e.g., K562, HL-60, NCI-H446, BEL7402, U251, HCT-8, and HeLa) [8]. It has also been reported that LDR does not induce adaptive responses in cancer cells *in vitro* or *in vivo* [8]. The distinct biological effects induced by LDR in normal and cancer cells suggest that specific mechanisms protect normal tissue against radiation-induced damage. However, few studies have directly compared the biological effects of LDR in normal and cancer cells under the same experimental conditions, and therefore the mechanistic basis for this difference remains unclear.

The fate of an irradiated cell is influenced by a complex and highly regulated signaling network [9–11], involving DNA damage repair and anti-oxidative mechanisms [9, 12–14]. Ataxia telangiectasia mutated (ATM) is a serine-threonine kinase of the phosphatidylinositol kinase-related kinase family that acts as an initial DNA damage sensor [15]. ATM phosphorylates more than 700 proteins involved in cell proliferation and cell cycle control, including AKT. In addition, ATM can be oxidized in the cytoplasm under oxidative stress independent of double-strand breaks (DSBs) [16] and functions as a redox sensor [17]. ATM deficiency causes defects in astrocyte proliferation by increasing cellular ROS levels, which can be partially rescued by N-acetyl-cysteine, suggesting that oxidized ATM maintains intracellular redox homeostasis and controls cell proliferation by phosphorylating components of some signaling pathways [18, 19]. Given that LDR can induce low levels of DSBs and ROS production, we hypothesized that it can activate ATM and its downstream effectors, which may account for the different biological effects of LDR in normal vs. cancer cells.

In the present study, we compared the effects of LDR on A549 lung adenocarcinoma cells and HBE135-E6E7 (HBE) normal lung epithelial cells with the focus on ATM and its associated signaling pathways. Our findings provide insight into the mechanism by which LDR protects normal cells against the damage of a subsequent HDR and suggest potential applications in anti-cancer treatment.

## RESULTS

### LDR stimulates cell proliferation and cell cycle progression of HBE cells but not of A549 cells

**Figure 1:**
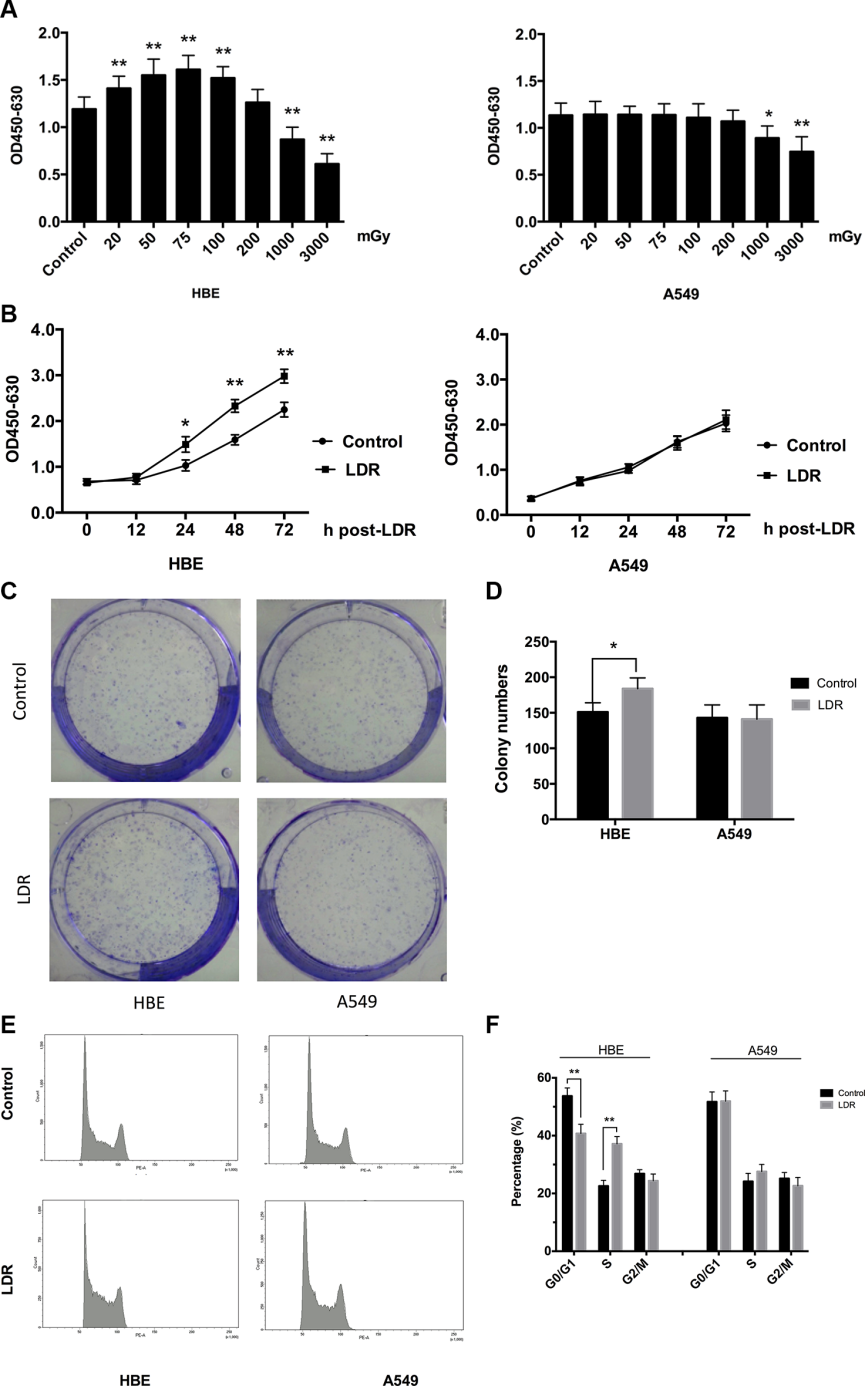
LDR stimulates cell proliferation and cell cycle progression of HBE cells but not A549 cells HBE (5 × 10^3^) and A549 (3 × 10^3^) cells were seeded in 96-well plates and irradiated with 20, 50, 75,100, 200, 1000, and 3000 mGy of X-ray. After irradiation, cells were transferred to an incubator and cultured for another 24 h. (**A**) Cell viability at indicated doses was determined with the WST-1 assay. (**B**) Cell viability was assessed at different times after exposure to 75 mGy X-ray with the WST-1 assay. (**C**) Representative images of colony formation of cells after exposure to 75 mGy X-ray. (**D**) Statistical analysis of colonies numbers in HBE and A549 cells 10 days after treated. (**E**) Cell cycle distribution after exposure to 75 mGy X-ray was analyzed 24 h post-LDR by flow cytometry. (**F**) Quantitative analysis of cells in each phase of the cell cycle. Data are presented as mean ± standard deviation of three separate experiments, with six replicates in each experiment. *P < 0.05 vs. control; **P < 0.01 vs.control.

The effects of LDR on mammalian cells are dose- and time-dependent [7]. We therefore examined the effects of different doses (20, 50, 75, 100, 200, 1000, and 3000 mGy) of X-rays on cell proliferation with WST-1 assay at 24 h after irradiation. The results showed that exposure to 20–100 mGy X-rays stimulated HBE cells proliferation relative to the control group, with the most significant effect observed at 75 mGy (Figure 1A, left panel); however, the proliferation of A549 cells was not affected within the dose range of 20–200 mGy (Figure 1A, right panel). When the radiation dose was increased to more than 200 mGy, the proliferation rate of both two types of cells was obviously declined. Then, proliferation rates of the two cell lines were examined at different time-points (0, 12, 24, 48, and 72 h) after irradiation at 75 mGy. HBE cells showed an accelerated proliferation between 24–72 h (Figure 1B, left panel), while there was no difference in A549 cell proliferation with or without irradiation (Figure 1B, right panel). Moreover, a classic clonogenic assays clearly demonstrate that LDR with 75 mGy X-rays increases the clonogenic survival of HBE cells but not of A549 cells (Figure 1C, 1D).

To confirm the pro-proliferative effect of LDR on HBE cells, cell cycle distribution at 24 h after 75 mGy irradiation was analyzed by flow cytometry. LDR caused a 1.67-fold increase in the S-phase fraction relative to control HBE cells (37.2% vs. 22.4%, *P* < 0.01), with a concomitant 1.32-fold decrease in the G0/G1 fraction (40.3% vs. 53.3%, *P* < 0.01) (Figure 1E, 1F, left panel). In contrast, we did not observe significant changes in A549 cell cycle distribution following irradiation (Figure 1E, 1F, right panel). These results consistently indicate that LDR at 75 mGy significantly induces hormesis in normal cells at 24 h post-LDR but not in cancer cells.

### ATM and its downstream target AKT are required for LDR-induced hormesis in HBE cells, but not in A549 cells

**Figure 2:**
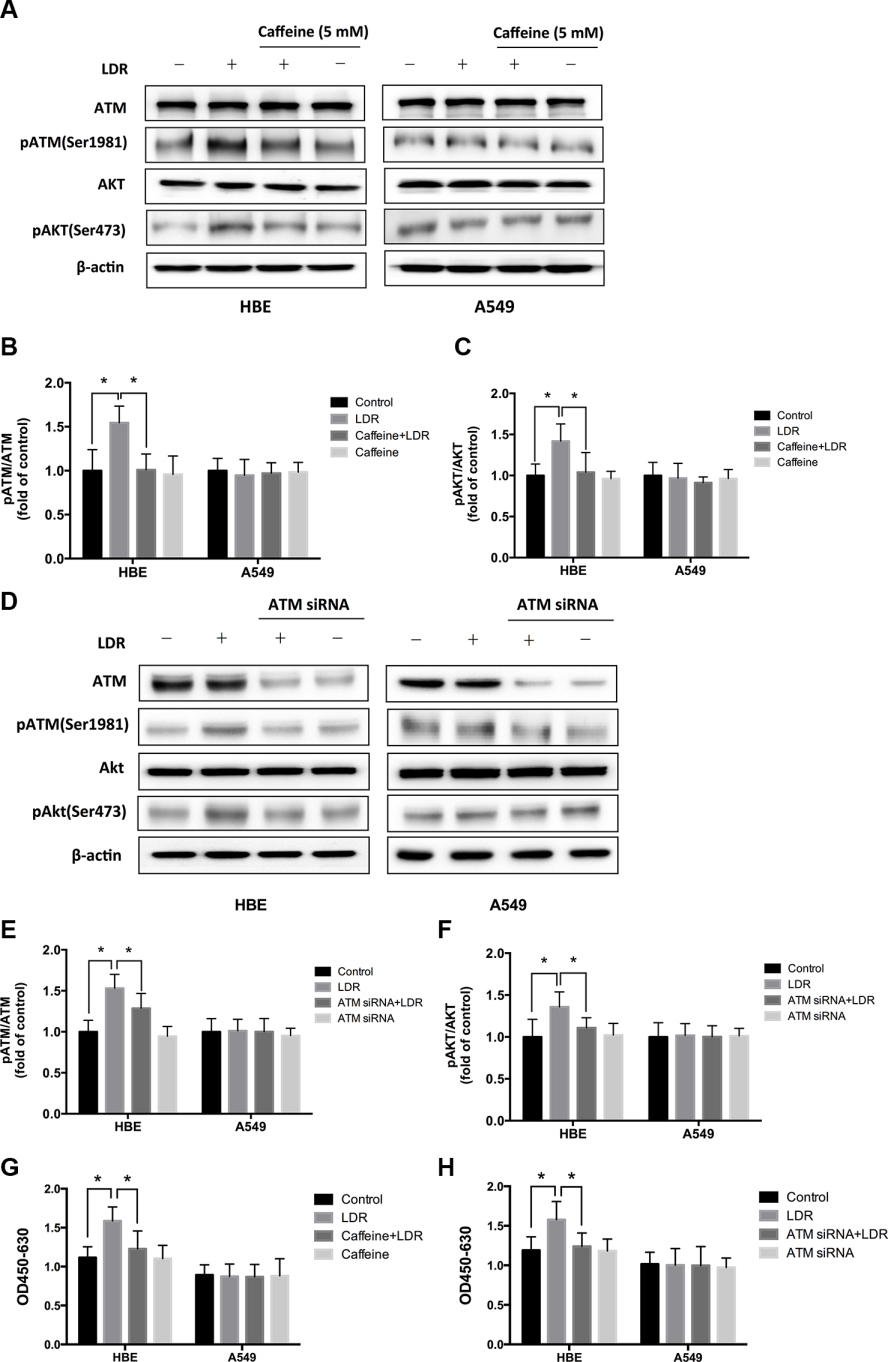
LDR induces ATM and AKT phosphorylation in HBE cells but not in A549 cells ATM inhibition blocked LDR-induced phosphorylation of ATM and AKT, and proliferation of HBE cells but not A549 cells. Cells were treated with 75 mGy X-ray, then transferred to an incubator and cultured for 24 h. For ATM inhibition, cells were pretreated with 5 mM caffeine or 25 nM ATM siRNA and then irradiated with 75mGy X-ray, then cultured for an additional 24 h before analysis. (**A**, **B**, **C**) ATM and AKT phosphorylation was assessed by western blotting and quantitative analysis in both HBE and A549 cell lines after LDR with or without pretreated with caffeine. (**D**, **E**, **F**) ATM and AKT phosphorylation were assessed by western blotting and quantitative analysis in both HBE and A549 cell lines after LDR with or without pretreated with ATM siRNA. (**G**) Cell viability was assessed with the WST-1 assay in both HBE and A549 cell lines after LDR with or without pretreated with caffeine. (**H**) Cell viability was assessed with the WST-1 assay in both HBE and A549 cell lines after LDR with or without pretreated with ATM siRNA. Data are presented as mean ± standard deviation of three separate experiments, with at least two of each sample per experiment. *P < 0.05.

The above finding that LDR stimulated proliferation and cell cycle progression in HBE cells but not in A549 cells suggests that it activated different signaling pathways in the two types of cells. We investigated whether the ATM pathway is involved in this process by evaluating the phosphorylation of ATM and its downstream target AKT by western blotting. X-ray irradiation at 75 mGy increased ATM and AKT phosphorylation in HBE cells relative to controls 24 h later (*P* < 0.05, Figure 2A–2F, left panel), without affecting total ATM and AKT protein levels. However, ATM and AKT phosphorylation were unaffected by LDR in A549 cells (*P* > 0.05, Figure 2A–2F, right panel).

To further assess the role of ATM and AKT in LDR-induced hormesis, we treated cells with caffeine (5 mM; 2 h), an ATM inhibitor, to block ATM function before irradiation. Caffeine inhibited LDR-induced ATM activation (*P* < 0.05, Figure 2A, 2B, left panel) and AKT phosphorylation (*P* < 0.05, Figure 2A, 2C, left panel) in HBE cells. Inactivation of ATM also inhibited the pro-proliferative effect of LDR in HBE cells (*P* < 0.05, Figure 2G, left panel). In contrast, caffeine treatment had no effect on A549 cells (*P* > 0.05, Figure 2A–2C, 2G, right panel). Furthermore, the requirement of ATM for LDR-induced hormesis was validated by transient transfection of HBE and A549 cells with ATM siRNA, which shows that LDR didn't simulate the phosphorylation of ATM in the presence of ATM siRNA in HBE cells (*P* < 0.05, Figure 2D, 2E, left panel). Consequently, the lack of AKT phosphorylation was also confirmed by western blotting analysis (*P* < 0.05, Figure 2D, 2F, left panel). In A549 cells, however, the activating status of ATM and AKT were still not altered by LDR regardless of the presence or absence of ATM siRNA (*P* > 0.05, Figure 2D–2F, right panel). Furthermore, transient ATM knockdown indeed abolished the proliferative effect of LDR in HBE cells (*P* < 0.05, Figure 2H, left panel). These data indicate that ATM/AKT signaling is required for LDR-induced hormesis in normal cells.

### LDR increases CDK4/CDK6/cyclin D1 expression via activation of ATM/AKT/GSK-3β signaling in HBE cells, but not in A549 cells

**Figure 3:**
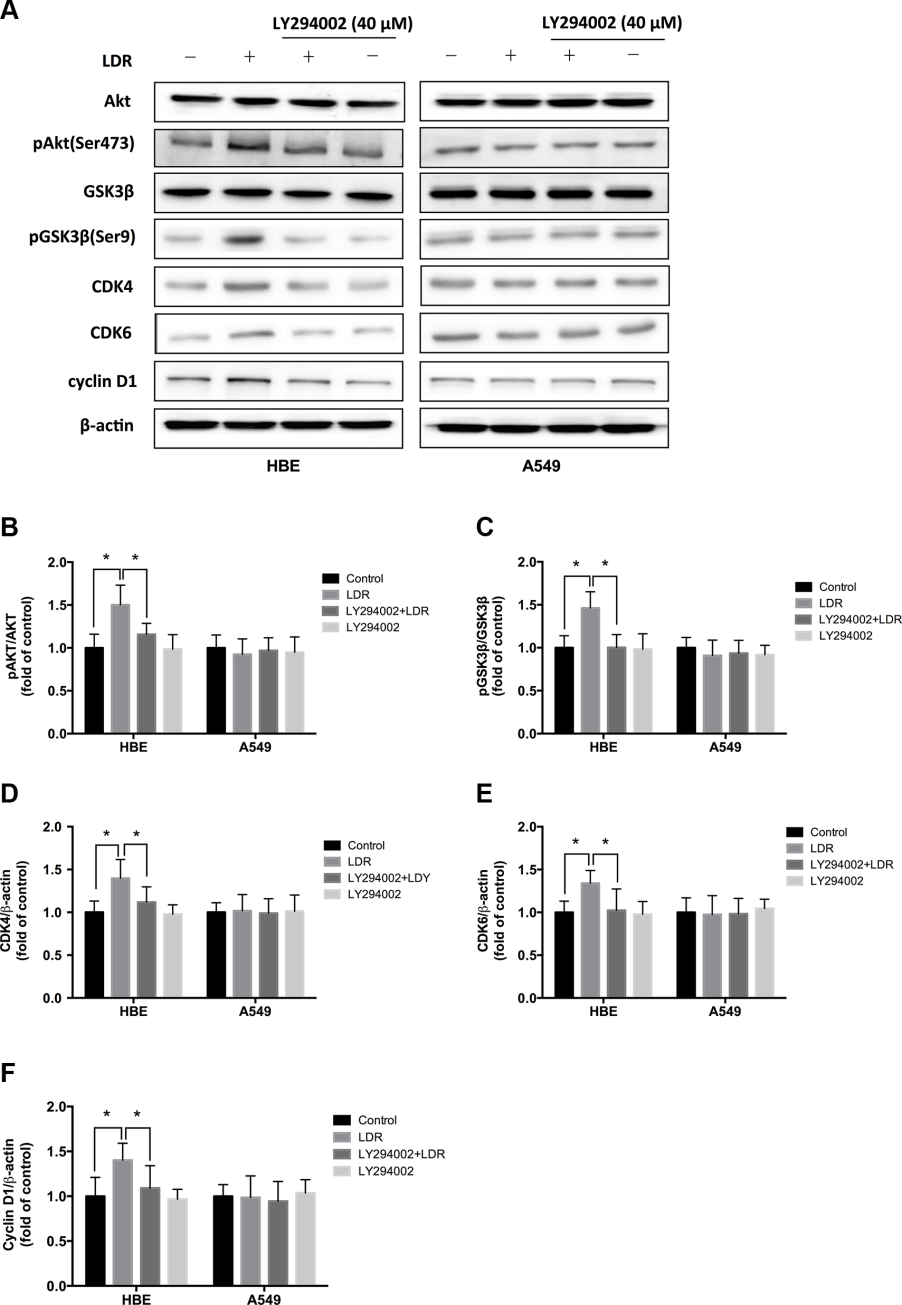
LDR increases GSK-3β phosphorylation and CDK4/CDK6/cyclin D1 expression in HBE cells but not in A549 cells For AKT inhibition, cells were pretreated with 40 μM LY294002 for 2 h, then irradiated at 75 mGy and cultured for an additional 24 h before analysis. (**A–F**) GSK-3β phosphorylation and CDK4/CDK6/cyclin D1 level were determined 24 h after irradiation with 75 mGy X-ray by western blotting and quantitative analysis in HBE and A549 cells with or without pretreated with LY294002. Data are presented as mean ± standard deviation of three separate experiments, with at least two of each sample per experiment. *P < 0.05.

LDR suppresses the proteolysis of cyclin D1 by inducing the constitutive activation of the ATM/AKT/glycogen synthase kinase (GSK)-3β signaling pathway [20]. As a regulatory subunit of cyclin-dependent kinase (CDK), cyclin D1 forms complexes with CDK4 and CDK6, which were a central mediator in the transition from G1 to S phase. Due to the induction of S-phase progression in HBE cells in response to LDR (Figure 1E, 1F) we examined the expression of CDK4, CDK6, and cyclin D1 by western blotting 24 h post-LDR as well as AKT and GSK-3β phosphorylation. Consistent with the observed LDR-induced increase in AKT phosphorylation in HBE cells, GSK-3β phosphorylation and CDK4, CDK6, and cyclin D1 expression were increased in the LDR as compared to the controls (*P* < 0.05, Figure 3A–3F, left panel). However, these effects were abolished along with the down-regulation of AKT phosphorylation in the HBE cells pretreated with the AKT inhibitor LY294002 (40 μM) 2 h prior to LDR exposure (*P* < 0.05, Figure 3A–3F, left panel). LDR had no effect on GSK-3β phosphorylation and CDK4, CDK6, and cyclin D1 expression in A549 cells regardless of the presence or absence of AKT inhibitor (*P* > 0.05, Figure 3A–3F, right panel). These results suggest that the activation of AKT/GSK-3β signaling induced by LDR increases CDK4/CDK6/cyclin D1 levels, leading to S-phase progression in normal cells.

### LDR increases mRNA and protein levels of Nrf2-dependent antioxidant factors in HBE cells but not in A549 cells

**Figure 4:**
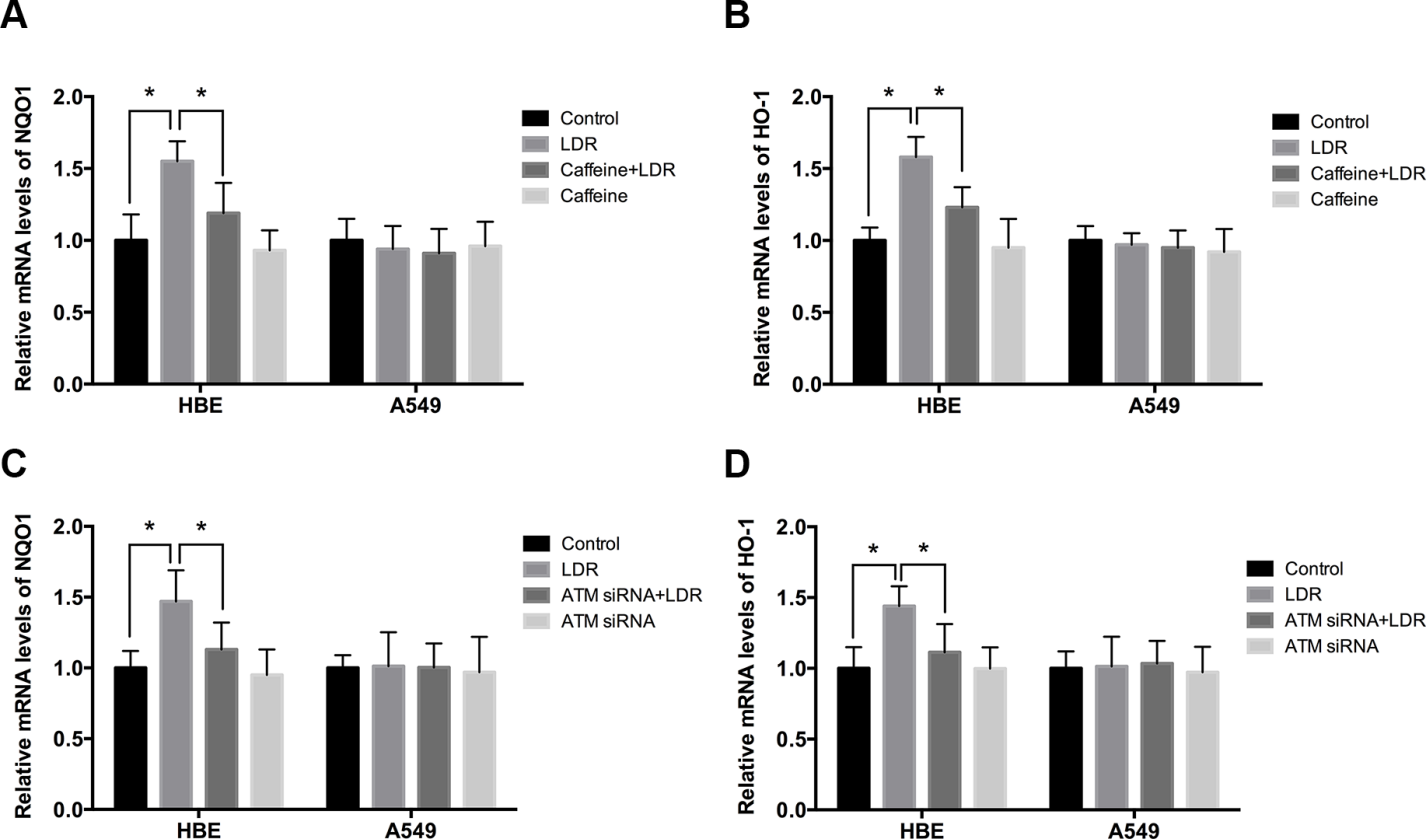
LDR increases transcript levels of Nrf2-dependent antioxidants via ATM-mediated signaling in HBE cells but not in A549 cells Cells were treated with 75 mGy X-ray, then transferred to an incubator and cultured for 24 h. For ATM inhibition, cells were pretreated with caffeine or ATM siRNA. (**A**–**D**) mRNA levels of *NQO1* and *HO-1* were assessed by real-time qPCR and normalized to that of β-actin, respectively. Data are presented as mean ± standard deviation of three separate experiments, with at least two of each sample per experiment. *P < 0.05.

**Figure 5:**
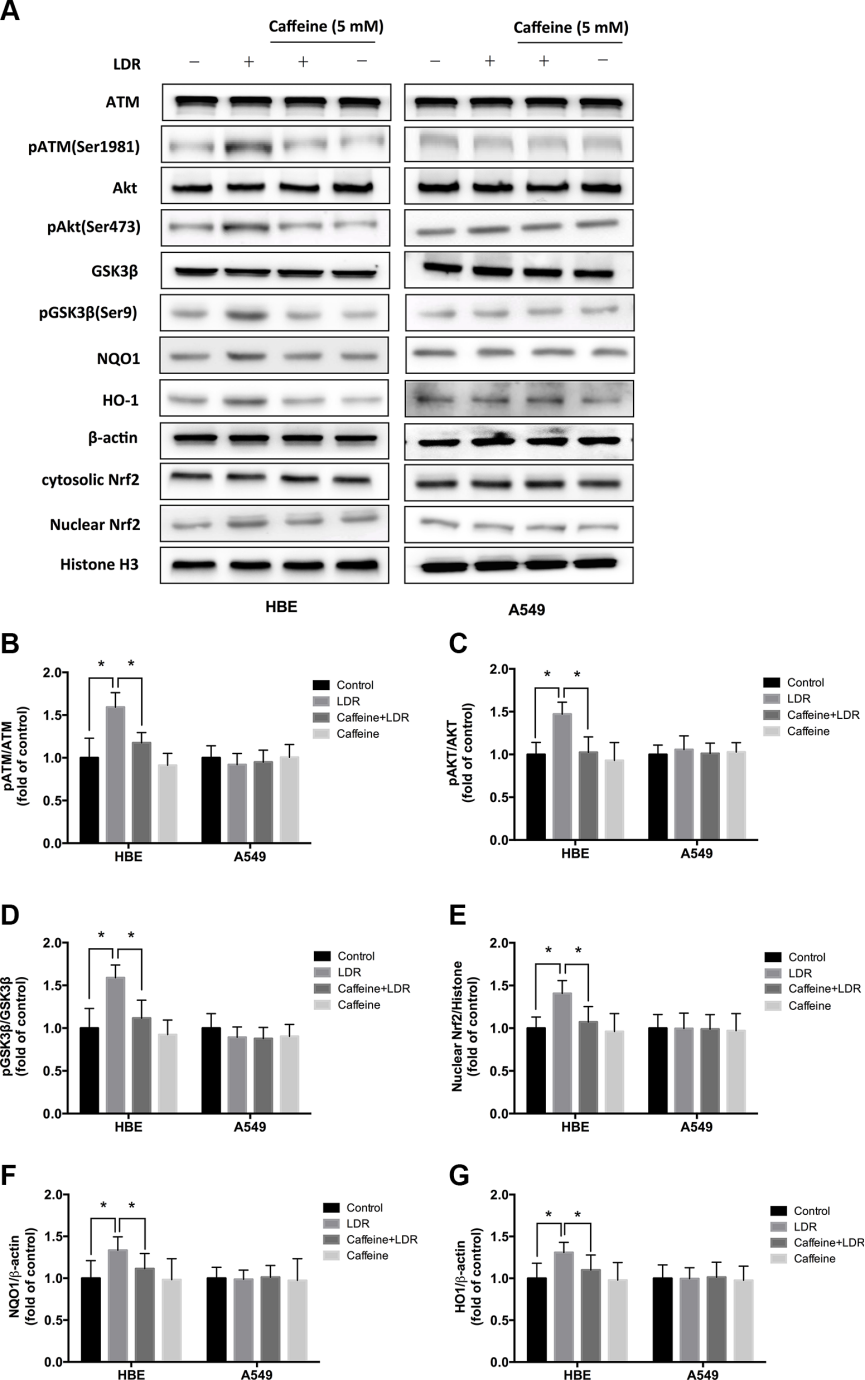
ATM inhibition with caffeine abolished LDR-induced phosphorylation of AKT and GSK-3β, nuclear accumulation of Nrf2, and expression of antioxidants in HBE cells but not in A549 cells Cells were pretreated with 5 mM caffeine for 2 h, then irradiated with 75 mGy X-ray and cultured for 24 h before analysis. (**A**–**G**) ATM, AKT, and GSK-3β phosphorylation, nuclear Nrf2, NQO1, and HO-1 level were determined by western blotting and quantitative analysis in HBE and A549 cells, respectively. Data are presented as mean ± standard deviation of three separate experiments, with at least two of each sample per experiment. *P < 0.05.

**Figure 6:**
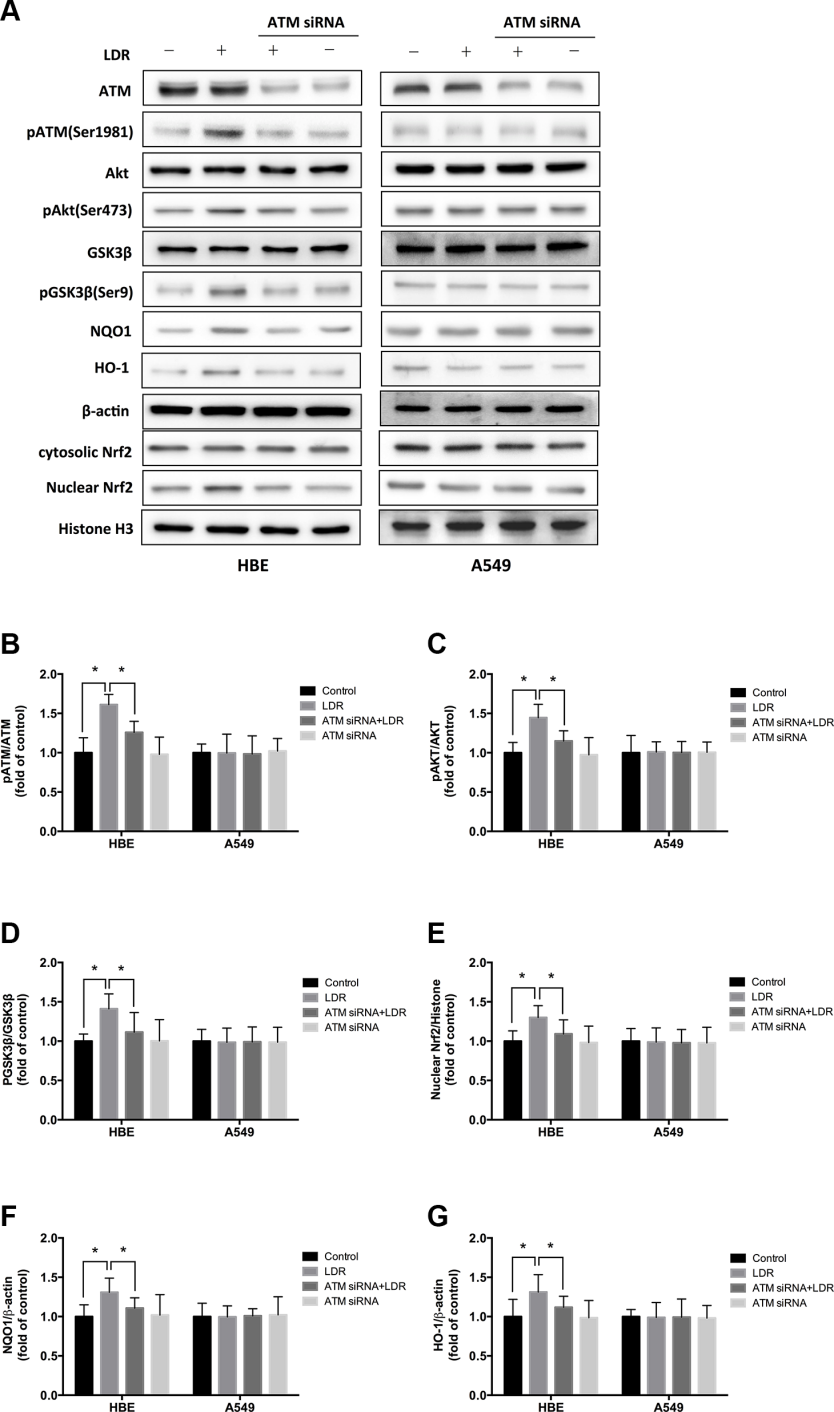
Knockdown of ATM with siRNA blocks LDR-induced phosphorylation of AKT and GSK-3β, nuclear accumulation of Nrf2, and expression of antioxidants in HBE cells but not in A549 cells For knockdown of ATM, cells were pretreated with 25 nM ATM siRNA. Twenty-four hours after transfection, cells were irradiated with 75 mGy X-ray and cultured for 24 h before analysis. (**A**–**G**) ATM, AKT, and GSK-3β phosphorylation nuclear Nrf2, NQO1 and HO-1 level were determined by western blotting and quantitative analysis in HBE and A549 cells, respectively. Data are presented as mean ± standard deviation of three separate experiments, with at least two of each sample per experiment. *P < 0.05.

It has been demonstrated that inactivation of GSK-3β can stimulate nuclear factor erythroid 2-related factor 2 (Nrf2) transcriptional activity, leading to upregulation of its downstream antioxidants [21, 22]. We therefore evaluated the transcript levels as well as protein levels of antioxidants (*NQO1* and *HO-1*) as downstream targets of Nrf2 in cells 24 h after LDR by real-time qPCR and western blot. In HBE cells, *NQO-1* and *HO-1* mRNA and protein levels in LDR groups were higher than those in control groups (*P* < 0.05), but there was no such difference observed in A549 cells (Figure 4A–4D; Figure 5A, 5F, 5G; Figure 6A, 6F, 6G). These findings suggest that LDR activates the antioxidant system in normal cells but not in cancer cells.

### ATM/AKT/GSK-3β signaling is required for LDR-induced Nrf2-dependent antioxidants in HBE cells, not in A549 cells

We examined whether LDR activates Nrf2 transcription and whether ATM/AKT/GSK-3β signaling plays a key role in this process. We found that LDR increased the level of Nrf2 in the nuclear fraction of HBE cells but not of A549 cells (Figure 5A, 5E; Figure 6A, 6E). This was accompanied by a significant increase in the phosphorylation levels of ATM, AKT, and GSK-3β in HBE cells; however, there were no changes in the phosphorylation status of these kinases in A549 cells under the same conditions (Figure 5A–5D; Figure 6A–6D). This suggests that the differential responses of the two cell types in response to LDR-induced Nrf2 activation are mediated by ATM and downstream effectors.

We therefore examined whether ATM/AKT/GSK-3β signaling acts as upstream of Nrf2-mediated antioxidants in response to LDR by pretreating cells with caffeine (5 mM; 2 h) or transfecting cells with ATM siRNA to inhibit ATM before LDR. ATM inhibition reduced the phosphorylation of AKT and GSK-3β as well as nuclear accumulation of Nrf2 in HBE cells (*P* < 0.05, Figure 5A–5E, left panel; Figure 6A–6E, left panel). Either caffeine or ATM siRNA suppressed the increase in NQO1 and HO-1 expression induced by LDR in HBE cells (*P* < 0.05, Figure 5A, 5F, 5G, left panel; Figure 6A, 6F, 6G, left panel). However, these LDR-induced effects were not observed in A549 cells (*P* > 0.05, Figure 5A–5G, right panel; Figure 6A–6G, right panel).

**Figure 7:**
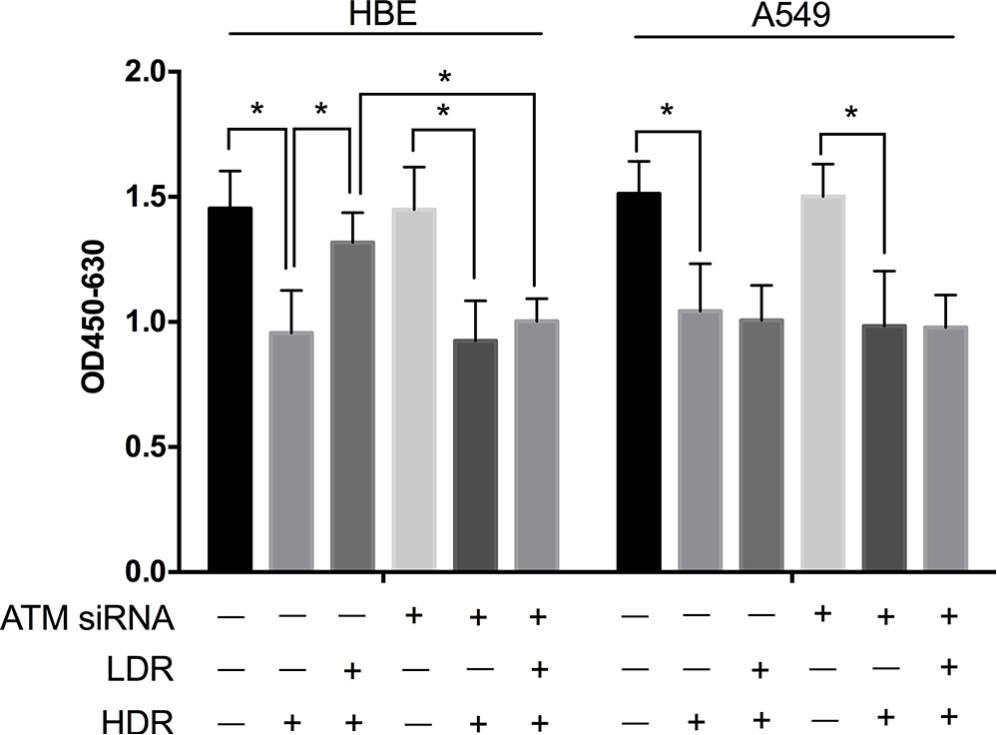
Knockdown of ATM with siRNA attenuated the protective effect of LDR in HBE cells but not in A549 cells Cells were pretreated with 25 nM ATM siRNA. Twenty-four hours after transfection, cells were exposure to LDR (75 mGy X-ray), cultured for another 24 h, and then treated by HDR (5 Gy X-ray) before analysis. Statistical analysis of the data from WST-1 assay showed that cellular viabilities of both HBE and A549 cells decreased drastically at 24 h after these cells were treated with HDR. Pretreatment with LDR protected HBE cells but not A549 cells against decrease in cell viability induced by HDR. However, knockdown of ATM attenuated significantly the protective effect of LDR against the cell viability decrease induced by HDR in HBE cells. Data are presented as mean ± standard deviation of three separate experiments, with at least two of each sample per experiment. *P < 0.05.

To verify the role of ATM in the protective effect of LDR in cells, we also examined the viability of LDR-pretreated HBE and A549 cells after HDR with or without knockdown of ATM. As shown in Figure 7, WST-1 assay proved that pretreatment with ATM siRNA significantly reduced the protective effect of LDR on HDR-induced cellular toxicity of HBE cells. These data collectively manifest a plausible relationship between the ATM/AKT/GSK-3β pathway and Nrf2-dependent antioxidant defense mechanisms that may ensure cell survival following exposure to LDR.

### LDR pretreatment protects HBE cells, but not A549 cells, against HDR-induced increases in intracellular ROS

**Figure 8:**
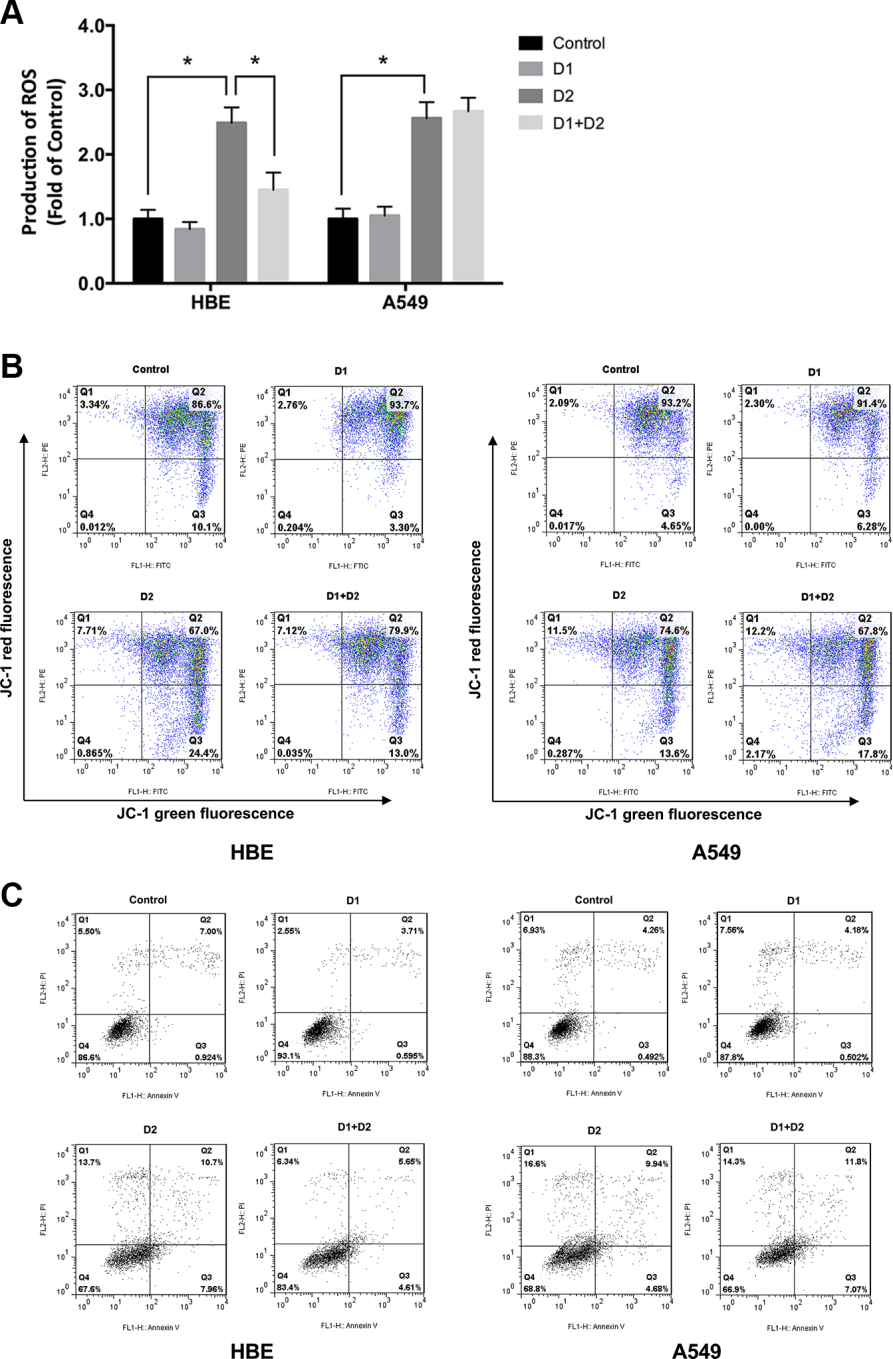
LDR pretreatment protects HBE cells but not A549 cells against increases in intracellular ROS induced by HDR Cells were divided into four groups: control (sham-irradiated), D1 (75 mGy), D2 (5 Gy), and D1 + D2 (75 mGy + 5 Gy). The interval between D1 and D2 was 24 h. Cells were analyzed after an additional 24 h of culture. (**A**) Intracellular ROS levels were analyzed with the oxidation-sensitive DCFH-DA probe and detected by flow cytometry. (**B**) Flow cytometry in combination with JC-1 staining showed that the mitochondrial decline caused by HDR in D2 group of HBE cells was significantly inhibited by pretreatment with LDR in D1 + D2 group. (**C**) The apoptosis rate of the four groups by flow cytometry showed that the increased apoptosis rate caused by HDR in D2 group of HBE cells was obviously attenuated by LDR pretreatment in D1 + D2 group. Data are presented as mean ± standard deviation of three separate experiments, with at least two of each sample per experiment. *P < 0.05.

It is known that HDR-induced increases in intracellular ROS levels or oxidative stress can be toxic to cells. The next question thus is whether ATM/AKT/GSK-3β-mediated Nrf2 activation of the antioxidants in response to LDR protects cells from HDR-induced oxidative stress/ROS accumulation. Cells were divided into four groups: control (sham-irradiated), D1 (75 mGy), D2 (5 Gy), and D1 + D2 (75 mGy + 5 Gy). Intracellular ROS were detected using the oxidation-sensitive 2′, 7′-dichlorodihydrofluorescin diacetate (DCFH-DA) probe. ROS levels were significantly increased in the D2 group as compared to the control in both HBE and A549 cells (*P* < 0.05, Figure 8A). However, ROS levels were significantly lower in the D1 + D2 as compared to the D2 group, in HBE cells, but not in A549 cells (Figure 8A).

As the production of ROS can affect the mitochondrial function and cell apoptosis status, we further measured the mitochondrial membrane potential (Δψm) with JC-1 and detected cell apoptosis rate by flow cytometry in above four groups, which may reflect the protective effects of LDR on cells. Quantitative analysis using flow cytometry revealed that the percentage of both HBE and A549 cells with loss of Δψ obviously increased in D2 group as compared with controls (HBE: Control 10.1% vs D2 24.4%; A549: Control 4.65% vs D2 13.6%; *P* < 0.05, Figure 8B). However, the loss trend of Δψm in response to D2 treatment was significantly reversed by pretreatment with D1 in HBE cells (D2 24.4% vs. D1 + D2 13.0%, *P* < 0.05, Figure 8B, left panel); but not in A549 cells (D2 13.6%vs. D1 + D2 17.8%, *P* > 0.05, Figure 8B, right panel). In addition, the cell apoptosis, assayed by flow cytometry, revealed the similar results to Δψm (Figure 8C). Based on the results, we speculate that LDR induces an adaptive response to subsequent HDR exposure by activating the antioxidant defense mechanism, which may explain the differential effects of LDR on normal and cancer cells.

## DISCUSSION

Conventional radiation therapy can lead to excessive production of ROS and DNA damage in normal tissues, leading to adverse effects and delays in the treatment process. Exposure to LDR has been shown to induce hormesis as well as an adaptive response to subsequent HDR in normal cells [23–25]. The dose range for normal cells used in the experiments of hormesis and adaptive response was from 5 to 200 mGy. In the same dose range of LDR, however, most studies have confirmed that the hormesis and adaptive response were absent in cancer cells [26, 27]. The difference of LDR-induced adaptive response in normal and cancer cells indicated its potential as a protective strategy in the clinical application of LDR in cancer therapy. It also inspired our interest to explore the molecular mechanisms underlying this difference for LDR-induced hormesis and adaptive response between normal and cancer cells. We report here that LDR-activated ATM is the initial event leading to hormesis in normal cells. ATM activation increased the expression of CDK4/CDK6/cyclin D1 via stimulation of the AKT/GSK-3β signaling pathway, resulting in the proliferation of HBE cells. The nuclear accumulation of Nrf2 was also increased by activation of ATM/AKT/GSK-3β signaling, which eventually increased antioxidant levels and mitigated damage from excessive ROS induced by HDR. These effects were not observed in A549 carcinoma cells, indicating that the failure to activate these pathways can account for the absence of LDR-induced hormesis and adaptive response in cancer cells.

LDR-induced hormesis is typically reflected by the stimulation of cell proliferation. In the present study, we confirmed that exposure to 20–100 mGy X-rays had a pro-proliferative effect in HBE cells, which was maximal at a dose of 75 mGy; however, the same effect in A549 cells was not induced in the dose range of 20–200 mGy. In contrast to HDR, we supposed that some cancer cells may be more resistant to LDR than normal cells.

ATM plays a critical role in the regulation of DNA damage response and cellular homeostasis. Low levels of DSBs resulted in increased AKT phosphorylation and triggered pro-survival signaling, which was dependent on ATM [28]. We found that exposure to LDR caused a significant increase in ATM and AKT phosphorylation in HBE cells but not in A549 cells. LDR induces low levels of DSBs or ROS, resulting in the activation of ATM, which stimulates cell proliferation via AKT-mediated pro-survival signaling. We proposed that the activation of ATM induced by radiation was dose-dependent and cell-type specific. In the same cell lines, the phosphorylation status of ATM after LDR was different from that after HDR. Dai et al. confirmed that the expression of ATM 1981Ser-P protein was not observed at 0 −200 mGy but at > 200 mGy γ-rays in A549 cells [27]. On the other hand, in the same dose range of LDR, different kinds of cells have different response pattern of ATM. We assumed that the difference of radiosensitivity between normal and cancer cells may be the cause of the different responses of ATM to LDR. Therefore, it is possible that the dose of LDR is insufficient to induce the levels of DNA lesions in cancer cells that can activate ATM protein kinase. Accordingly, our study was to explore the possible molecular mechanism mediated by ATM by which LDR protects normal cells but not cancer cells against the damage caused by a subsequent HDR and suggest potential applications in cancer treatment.

GSK-3β is a downstream target of AKT and MAPK kinases; the activation of these pathways inhibits GSK-3β via phosphorylation at Ser9 [29], thereby decreasing its kinase activity and preventing cytoplasmic proteasomal degradation of cyclin D1 [30, 31]. We conclude that ATM/AKT signaling positively regulates CDK4/CDK6/cyclin D1 expression by inactivating GSK-3β, resulting in G1/S cell cycle progression of HBE cells but not A549 cells in response to LDR. The effect of activated ATM on cell cycle is usually affected by multiple factors, including cell types, cell cycle status, effectors of ATM and so no. It is known that ATM modulates many effectors, which involve in cell proliferation and cell cycle control. The crosstalk of these effectors may lead to different fate of cells. Some studies demonstrated that the activated ATM protein could regulate multiple nuclear and cytoplasmic events leading to cell cycle arrest [32, 33]. However, different results were also reported. Shimura et al. demonstrated that a moderate level of long-term fractionated radiation induces ATM-mediated cyclin D1 overexpression, which leads to forced progression of the cell cycle to S-phase [34]. Another study by Tang et al. reported that the activated ATM protein kinase promoted the proliferation of cancer-associated fibroblasts, which was shown as a faster cell growth rate, and more cells in S-phase [35]. Similar with these results, we found that LDR stimulated the proliferation of HBE cells and promoted the S-phase progression via ATM-mediated signal pathway. In addition, we supposed that the variation in cell cycle affected by ATM reported among the different studies could be related to cell type. In addition to HBE and A549 cells, we also detected the effect of LDR on the proliferation and cell cycle of other cells, which showed that the S-phase of prostate cancer cells was arrested after LDR treatment, accompanied by the declined proliferation rate (Yu et al. accepted by Int. J. Mol. Med.). Collectively, our findings supports the hypothesis that activation of the ATM/AKT/GSK-3β signaling pathway leading to CDK4/CDK6/cyclin D1 overexpression plays a key role in LDR-induced hormesis, and is responsible for the difference of hormesis induced by LDR in normal and cancer cells.

Radiation doses that cause hormesis are also effective in inducing an adaptive response [36, 37]. In present study, we found that pretreatment with LDR at 75 mGy reduced HDR-induced intracellular ROS levels and mitochondrial decline in HBE cells but not in A549 cells, suggesting that activation of anti-oxidative mechanisms plays an important role in the LDR-induced adaptive response.

ATM-dependent activation of AKT/GSK-3β/Nrf2 signaling and increased antioxidant expression were identified in LDR-induced anti-oxidative response, which was further supported by the fact that blocking ATM with caffeine or ATM siRNA inhibited AKT phosphorylation, GSK-3β phosphorylation as well as Nrf2 nuclear accumulation. Nrf2 directly regulates the expression of most antioxidants and is critical for the LDR-induced anti-oxidative response [38]. AKT can be activated by phosphorylation at Ser473 [39], which phosphorylates GSK-3β at Ser9, thereby regulating Nrf2 expression and function [40, 41]. Although direct evidence is lacking, a number of studies have suggested that ATM is closely linked to Nrf2-mediated antioxidant signaling [42, 43]. Our results provide the new evidence that an ATM-initiated signaling pathway participates in Nrf2-mediated anti-oxidative response.

Moreover, it has been reported that LDR could induce a rapid and transient elevation of ROS (such as 30 min or 1 h post-irradiation) in normal cells [44, 45]. In our study, it has shown that LDR activated Nrf2-mediated antioxidant system through ATM-related signaling pathway. Thus, we hypothesized that the concentration of ROS in HBE cells decline slightly at 48 h after LDR, due to the subsequent elimination of the elevated ROS by activation of antioxidant response. At the same time, it is demonstrated that the proliferation and ATM phosphorylation increase in HBE cells after exposure to LDR. But in A549 cells, it doesn't show any change. Therefore, the proliferation results of both HBE and A549 cells after LDR were consistent with the phosphorylation status of ATM, respectively.

Additionally, since cells in G2/M phase are more sensitive to radiation, an increase in the G1/S-phase fraction induced by LDR in HBE cells may lead to resistance of cells to HDR, which indicates that ATM-dependent pro-survival signaling may also play a role in the difference of LDR-induced adaptive response between normal and cancer cells. Our results using A549 cells differed from those reported by a recent study that showed 50 mGy α-particle radiation conferred radio-resistance in A549 cells via activation of the Nrf2/HO-1 antioxidant pathway [46]. We speculate that the discrepancy may be due to differences in linear energy transfer (LET) and time points of observation. X-rays (low LET) and charged particles (high LET) at the same dosages induce different biological effects [47]. In addition, we considered that the results of our study were not contradictory to those in the study by Saskia et al [48]. The data in that study suggested that LDR with 40–80 mGy induced increase of γ-H2AX focus in human fibroblasts, which were activated by ATM kinase and made cells harboring the DNA repairing ability. In our study, we used the dose of 75 mGy in the experiments of LDR-induced protective response, which was within the dose range of 40–80 mGy. We also supposed that LDR could induce low levels DSBs or ROS that lead to the activation of ATM kinase through auto-phosphorylation and activation of DNA repair pathways and other cellular protective response. However, our present study is more focus on the LDR-induced antioxidant protective response. So we didn't examine the formation of γ-H2AX foci after LDR in the present study. Moreover, unlike in the previous study, we compared the biological effects of LDR on normal lung epithelial cells and cancer cells, revealing differences between two cell types that have clinical significance in cancer treatment. Nonetheless, the discordance between these findings highlights the complexity of the effects induced by LDR, which warrants further examination.

**Figure 9:**
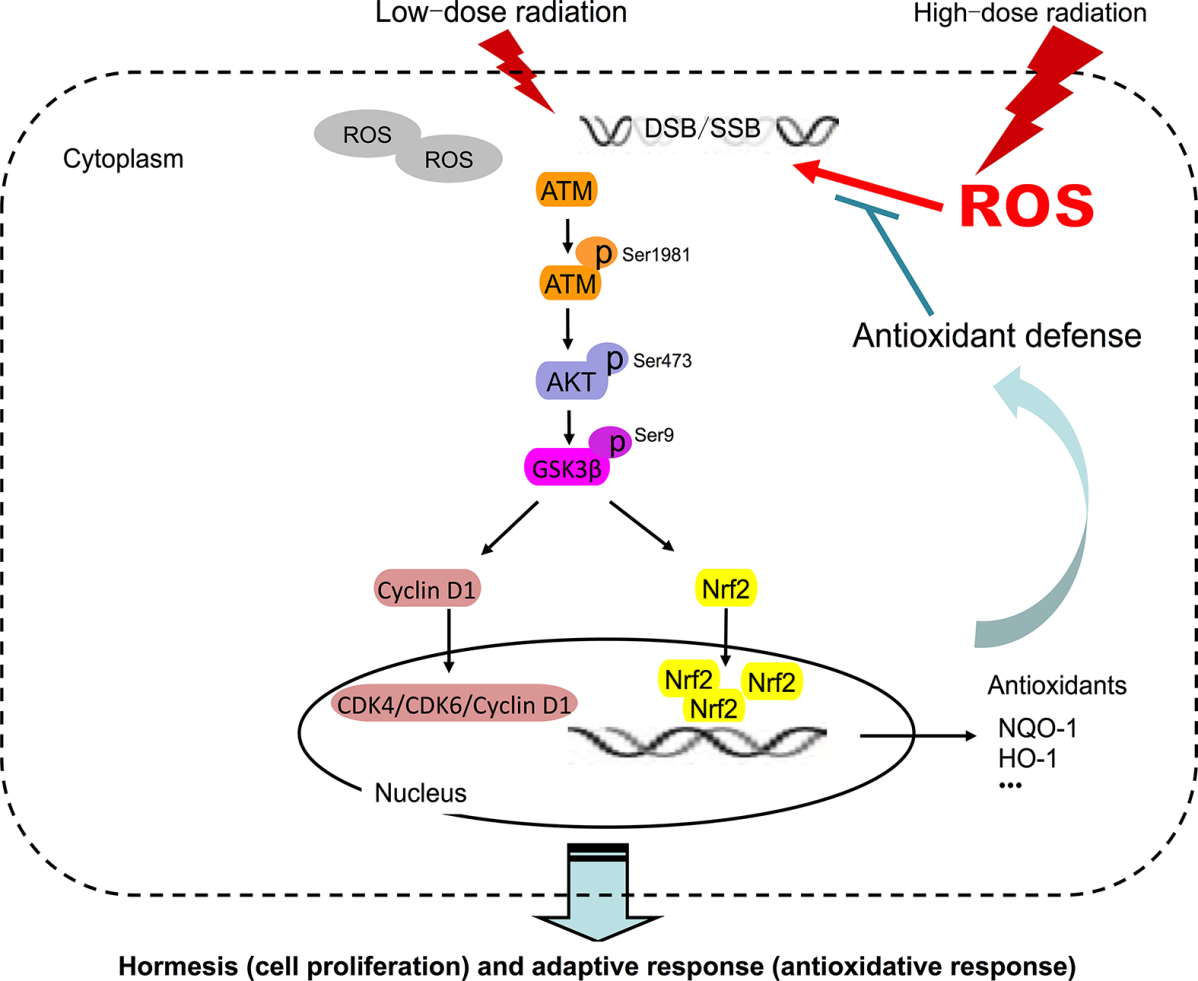
Model of LDR-induced hormesis and adaptive response in normal cells but not in cancer cells DSB, double-strand break; *p*, phosphorylated; ROS, reactive oxygen species; SSB, single-strand break.

In summary, our results suggest that LDR induces hormesis and adaptive response in normal lung epithelial cells but not in lung cancer cells. This effect was associated with ATM-mediated pro-survival and anti-oxidative mechanisms. In normal cells, ATM activation by LDR was the initial event that caused hormesis by increasing the expression of CDK4/CDK6/cyclin D1 via activation of AKT/GSK-3β signaling; the adaptive response involved the nuclear accumulation of Nrf2 (Figure 9). Our study identifies a plausible mechanism to explain the distinct effects induced by LDR in normal and cancer cells. In addition, our findings suggest that LDR may serve as an effective and safe therapeutic approach for the protection of normal tissue against damage caused by conventional radiotherapy.

## MATERIALS AND METHODS

### Cell culture

A549 human lung adenocarcinoma and HBE135-E6E7 human lung epithelial cells were purchased from the American Type Culture Collection (Manassas, VA, USA). A549 cells were maintained in high-glucose Dulbecco's Modified Eagle's Medium (DMEM) supplemented with 10% fetal bovine serum and 1% antibiotics (penicillin-streptomycin) (Invitrogen, Thermo Fisher Scientific, Waltham, MA, USA). HBE cells were maintained in keratinocyte serum-free medium supplemented with 5 ng/ml human recombinant epidermal growth factor, 0.05 mg/ml bovine pituitary extract, 0.005 mg/ml insulin, 500 ng/ml hydrocortisone, and 1% antibiotics (all from Invitrogen). Cells were cultured at 37°C in a humidified incubator with a constant airflow of 5% CO_2_. For ATM and AKT inhibition, cells were treated with caffeine (5 mM) (Sigma, Shanghai, China) solubilized in distilled water and LY294002 (40 μM) (Merck KGaA, Darmstadt, Germany) solubilized in dimethylsulfoxide for 2 h prior to irradiation. Media containing the inhibitors were replaced with fresh medium immediately after irradiation.

### X-ray irradiation

In the hormesis study, cells were irradiated with 20, 50, 75, 100, 200, 1000, and 3000 mGy X-rays at room temperature. In the adaptive response study, cells were exposed to an optimum adaptive dose (D1) that was confirmed in the hormesis study, with or without a challenge dose (D2) of 5 Gy. The interval between D1 and D2 was determined based on the results of the WST-1 assay and cell cycle analysis. Control groups were treated in a similar manner but without irradiation. After an additional 24 h of incubation, cells in each group were collected for analysis. Cells were irradiated using an X-RAD 320 X-Ray Biological Irradiator (Precision X-Ray, North Branford, CT, USA). Dose rates of 12.5 mGy/min and 500 mGy/min were used for LDR and HDR, respectively.

### Cell viability assay

A549 (3 × 10^3^) and HBE (5 × 10^3^) cells were seeded in a 96-well plate and irradiated 24 h later with indicated dose, then immediately transferred to an incubator and cultured for indicated time. Cell viability was evaluated at these doses and at different times after exposure to the indicated dose with the WST-1 assay (Roche, Shanghai, China) according to the manufacturer's instructions. Absorbance was measured with a microplate reader (Bio-Rad, Hercules, CA, USA) at 450–630 nm. Each experiment was independently carried out three times with a total of 6 replicates in each experiment.

### Clonogenic assay

The clonogenic assay was performed as described previously [49]. Briefly, cells were plated in six-well plates and were exposed to LDR. After irradiation, the cells were cultured for 10 days. After washed and fixed with ethanol and stained with crystal violet, cell colonies (defined as a colony with > 50 cells) were counted under a microscope. Three independent experiments were performed.

### Cell cycle and cell apoptosis analysis

Around 2 × 10^6^cells were collected at indicated time after being treated with indicated irradiation dose. Cell pellets were fixed with ice-cold 70% ethanol in PBS at −20°C for at least 2 h and then centrifuged at 1,500 rpm for 5 min. The pellet was incubated with 0.5% Triton X-100 (Sigma) and 0.05% RNase (Sigma) in 1 ml PBS at 37°C for 30 min. For cell cycle analysis, cell pellets were resuspended in 400 μl PBS containing 40 μg/ml propidium iodide (PI, Sigma) and incubated for 30 min in dark at room temperature. For cell apoptosis, cells were washed with 1 × PBS (4°C), incubated with 400 μl 1 × binding buffer containing 5 μl Annexin V-FITC and 5 μl with PI for 30 min in dark at room temperature. Samples were immediately analyzed by a FAC-Scan flow cytometry (BD Biosciences, Franklin Lakes, NJ, USA). The distribution of cell cycle and the variation of cell apoptosis were analyzed using FlowJo software (Tree Star, Inc. Ashland, OR, USA). Three independent experiments were performed.

### Determination of intracellular ROS levels

DCFH-DA was used to analyze intracellular ROS levels. Cells were detached by trypsinization and washed twice with PBS, then incubated in 300 μl cell culture medium containing 20 μM DCFH-DA for 30 min at 37°C. ROS fluorescence was measured at an excitation and emission of 488 and 525 nm, respectively, using a Synergy 4 microplate reader (BioTek Instruments, Winooski, VT, USA). The fluorescence of treated cells was compared to that of the appropriate controls. Experiments were performed with triplicate samples and data are representative of at least three independent experiments.

### Mitochondrial membrane potential (JC-1) assay

Using flow cytometry to detect the 5,5′,6,6′-tetrachloro-1,1′,3,3′-tetraethylbenzi- midazolyl- carbocyanine iodide (JC-1) (Beyotime Biotech, Shanghai, China). After incubation with the JC-1 staining solution at 37°C in the cell incubator for 10 min, cells were washed with JC-1 staining buffer two times and then analyzed by flow cytometry. In the normal mitochondria, JC-1 aggregate to form a polymer in the mitochondrial matrix, the polymer sends a strong red fluorescence (Ex = 585 nm, Em = 590 nm); While in the unhealthy mitochondrial, due to the decline or loss of the mitochondrial membrane potential, JC-1 monomers just can be present in the cytoplasm, resulting in a green fluorescence (Ex = 514 nm, Em = 529 nm). Therefore, using flow cytometry to observe the color changes reflects very directly the early change of mitochondrial membrane potential.

### siRNA transfection

The sequence of human ATM siRNA (sc-29761) and control siRNA (sc-37007) were from Santa Cruz Biotechnology. HBE and A549 cells were seeded in 6-well plates at a density of 2 × 10^5^/well in 2 ml cell culture medium with 10% FBS, respectively. When the cell density reached 60–70% confluence, cells were washed twice with 2 ml Opti-MEM Reduced Serum Medium. Transfection with 25 nM siRNAs was performed using Lipofectamine^®^ 2000 Transfection Reagent (Invitrogen, Thermo Fisher Scientific, Waltham, MA, USA) in agreement with the manufacturer's instruction. Twenty-four h after transfection, cells were divided into each indicated group for subsequent experiments.

### Real-time qPCR

Total RNA was extracted with TRIzol reagent (Invitrogen), and RNA concentration and purity were determined using a Nanodrop ND-1000 spectrophotometer (Invitrogen). cDNA was synthesized from total RNA using Moloney murine leukemia virus reverse transcriptase (Invitrogen). Real-time qPCR was carried out in a 10-μl reaction volume containing 5μl SYBRRT-PCR Master Mix, 1 μl each primer, and 4 μl cDNA using a CFX384 Touch Real-Time PCR Detection System (Bio-Rad, CA, USA). Each sample was prepared in triplicate. Primers for *NQO1*, *HO-1*, and *β-actin* (internal control) were purchased from Comate Bioscience Co. (Changchun, China). The fluorescence intensity of each sample was measured at each temperature change to monitor amplification of the target gene. The comparative cycle threshold (2^−ΔΔCt^) method was used to determine the amount of target normalized to the internal reference relative to a calibrator.

### Western blot analysis

Cells were washed twice with ice-cold PBS, collected in PBS using a rubber scraper, and centrifuged at 1000 rpm for 5 min. Biochemical fractionation was carried out using a nuclear extraction kit (Active Motif, Carlsbad, CA, USA) according to the manufacturer's instructions. For making total cell lysate, cells were lysed in radio-immunoprecipitation buffer supplemented with 1 mM phenylmethylsulfonyl fluoride (Beyotime Biotechnology, Shanghai, China). Protein concentration was determined with an enhanced bicinchoninic acid protein assay kit (Beyotime Biotechnology). Protein samples (40–60 μg) were separated by 8% or 12% sodium dodecyl sulfate-polyacrylamide gel electrophoresis and transferred to a polyvinylidenedifluoride membrane. Following 1–2 h of incubation in fresh Tris-buffered saline buffer containing 0.1% Tween-20 and 5% bovine serum albumin, the blots were probed with primary antibody overnight at 4°C. Bound primary antibodies were detected with horseradish peroxidase-conjugated anti-rabbit or -mouse IgG. Peroxidase activity was visualized by enhanced chemoluminescence (Thermo Fisher Scientific). Mean band intensity was determined by densitometry using ImageJ software (National Institutes of Health, Bethesda, MD, USA). Primary antibodies against the following proteins were used: AKT, phospho-AKT (Ser473), GSK-3β, phospho-GSK-3β (Ser9), CDK4 and CDK6 (Cell Signaling Technology, Danvers, MA, USA); ATM, phospho-ATM, and cyclin D1 (Epitomics, Burlingame, CA, USA); Nrf2 (Santa Cruz Biotechnology, Santa Cruz, CA, USA); NQO1 and HO-1 (Abcam, Cambridge, MA, USA). Antibodies against β-actin and histone H3, and anti-rabbit and -mouse IgG were purchased from Beyotime Institute of Biotechnology.

### Statistical analysis

Data were collected from repeated experiments and expressed as means ± standard deviation. Differences between groups were analyzed using the Student's *t* test, with GraphPad Prism v6.0 software (GraphPad Software, Inc., California, USA). *P* values < 0.05 were considered statistically significant.
